# Pulmonary schistosomiasis mimicking IgG4‐related lung disease

**DOI:** 10.1002/rcr2.276

**Published:** 2017-10-16

**Authors:** Timothy Baird, Caroline L Cooper, Richard Wong, Naomi Runnegar, Gregory Keir

**Affiliations:** ^1^ Department of Respiratory and Sleep Medicine Princess Alexandra Hospital Brisbane Australia; ^2^ Department of Anatomical Pathology, Pathology Queensland Princess Alexandra Hospital Brisbane Australia; ^3^ Southside Clinical Unit, Faculty of Medicine University of Queensland Brisbane Australia; ^4^ Department of Immunology, Princess Alexandra Hospital & Division of Immunology Pathology Queensland Central Laboratory, Royal Brisbane and Women’s Hospital Brisbane Australia; ^5^ Department of Infectious Diseases Princess Alexandra Hospital Brisbane Australia

**Keywords:** IgG4‐related disease, lung, schistosomiasis

## Abstract

IgG4‐related disease (IgG4‐RD) is a systemic fibroinflammatory disease characterized by tumefactive lesions in various organ systems, including the lungs. Clinical and radiological manifestations of IgG4‐RD are relatively non‐specific, and we report a case highlighting the importance of histopathological confirmation in cases of suspected IgG4‐related lung disease. A 44‐year‐old male with significantly elevated serum IgG4 levels, patchy consolidation on thoracic CT imaging, and cough was referred with suspected IgG4‐related lung disease. However, surgical lung biopsy revealed an unexpected diagnosis of pulmonary schistosomiasis, and following treatment with praziquantel, cough resolved and IgG4 levels significantly declined. This case highlights the potentially diverse array of conditions that may mimic IgG4‐related lung disease and the importance of comprehensive evaluation including histopathological confirmation where possible.

## Introduction

IgG4‐related disease (IgG4‐RD) is a systemic fibroinflammatory disease characterized by elevated serum IgG4 levels and typical histological features, including a lymphoplasmacytic inflammatory tissue infiltrate rich in IgG4‐positive plasma cells and varying degrees of tissue fibrosis 1. Originally described as affecting the pancreas (so‐called sclerosing pancreatitis), IgG4‐RD is now recognized as a potentially multisystem disease that may involve the gallbladder, thyroid, retroperitoneum, kidneys, and a variety of other organs 1, 2. IgG4‐related lung disease is increasingly recognized with reported manifestations including mediastinal adenopathy, interstitial involvement, parenchymal nodules, and consolidation 3. Respiratory symptoms are typically non‐specific, including cough, dyspnoea, chest pain, and fever. Given the non‐specific nature of symptoms and diverse radiological findings, a definitive diagnosis of IgG4‐related lung disease often requires histopathological confirmation and clinico‐pathological correlation.

We report a case of a 44‐year‐old male referred with suspected IgG4‐related lung disease that highlights the importance of a comprehensive clinical assessment, including a confirmatory tissue biopsy. Despite elevated serum IgG4 levels coupled with suggestive symptoms and radiological changes, a surgical lung biopsy revealed an unexpected diagnosis of pulmonary schistosomiasis.

## Case Report

**Figure 1:**
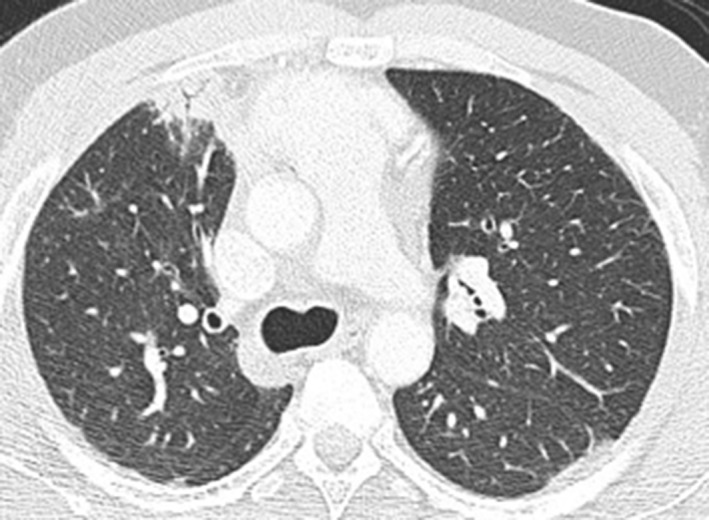
CT imaging demonstrating nonspecific patchy sub‐pleural consolidation anteriorly in the right upper lobe and in the apical segment of the left lower lobe.

A 44‐year‐old Zimbabwean‐born male was referred with a 12‐month history of cough, areas of patchy sub‐pleural consolidation on thoracic Computed Tomography (CT) (Fig. 1), and a significantly elevated serum Immunoglobulin subclass 4 (IgG4) level (4.61 g/L; normal range 0.08–1.4 g/L). He was a lifelong non‐smoker with no significant prior medical history and, particularly, no history of pancreatitis. He was not on any regular medications. Physical examination was unremarkable, and lung function testing revealed an isolated mild reduction in forced vital capacity (3.3 L, 78% predicted) with normal gas transfer. Connective tissue disease and vasculitic serology was negative, and C‐reactive protein (CRP) was 2.7 mg/L (normal <5 mg/L). Absolute blood eosinophil count of 0.47 X 10^9^/L (normal <0.60 X 10^9^/L), however, was elevated as a proportion of white cells at 8%.

**Figure 2:**
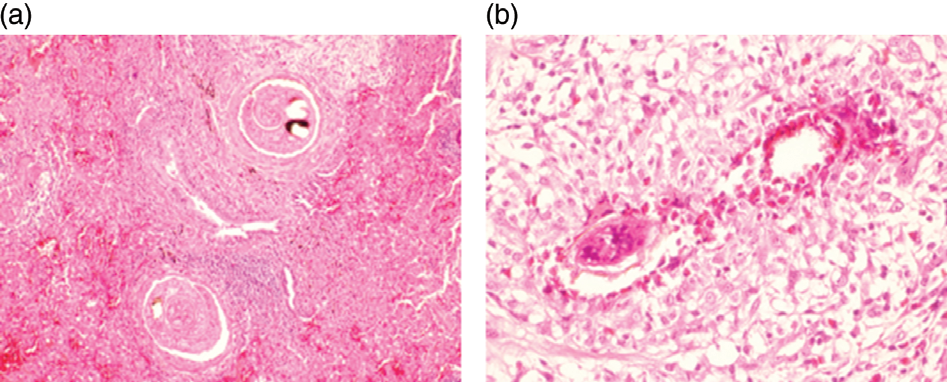
Histopathology of right upper lobe wedge biopsy. (A) Adult worms within blood vessels (H&E, 100×) and (B) granulomatous inflammation with numerous eosinophils surrounding schistosome eggs (H&E, 200×).

Bronchoscopy with bronchoalveolar lavage and trans‐bronchial lung biopsy was non‐diagnostic, and he proceeded to a surgical lung biopsy. Unexpectedly, this demonstrated adult schistosome worms within the pulmonary vessels and eggs with a florid granulomatous inflammatory reaction within the interstitium (Fig. 2). Subsequent stool microscopy confirmed the presence of *Schistosoma mansoni* ova; microscopy of urine did not reveal any schistosome ova. A screening echocardiogram revealed normal pulmonary artery pressures with no evidence of pulmonary hypertension.

He was treated with praziquantel at a total dose of 40 mg/kg (3600 mg), split into two doses administered 4 h apart. At three‐ and 12‐month follow up, his cough had resolved, and serum IgG4 levels had reduced significantly (from 4.61 to 1.59 g/L). Stool microscopy became negative for schistosome ova, and the eosinophil percentage of total white cells normalized (4%). Repeat CT imaging 12 months after eradication treatment revealed unchanged patchy consolidation, consistent with localized fibrotic pulmonary change.

## Discussion

In patients with suspected IgG4‐related lung disease, our case highlights the importance of histopathological confirmation given the non‐specific symptoms and radiology and the variety of causes for elevated serum IgG4 levels (including vasculitides, connective tissue disorders, infections, and malignancy) 1.

Although cases of IgG4‐related lung disease are increasingly reported, the underlying pathogenesis of the disease remains poorly understood. Hypothesized mechanisms suggest either an allergic‐type reaction with overexpression of Th2‐related cytokines, or as a result of circulating B cells entering inflamed tissue with subsequent differentiation and proliferation into IgG4‐switched B cells and, subsequently, plasma cells 1. Of particular relevance to our case, it has been well described that helminth infections demonstrate Th2 immune responses in addition to two recent cases of paragonimiasis presenting with specific elevated serum IgG4 levels 4, 5. These cases, in combination with our report, support the proposal that the underlying pathogenesis of IgG4‐RD may involve exposure to a particular antigen that leads to specific Th2 immune response with subsequent class switching towards IgG4 production by B and plasma cells.

Schistosomiasis is an endemic parasitic infection seen mainly in tropical regions, with humans infected as a result of schistosoma entering the circulation via the skin during contact with fresh water. The circulating parasites then pass through the heart, lungs, and liver to reach their target venous plexus where the adult worms mature, mate, and eventually release eggs into the environment 5. This cycle can result in various acute, subacute, or chronic disease states as a result of host immune responses, focal granulomatous inflammation, and fibrosis, in addition to the development of chronic disease states including hepatosplenomegaly with portal hypertension and obliterative pulmonary arteritis with pulmonary hypertension 5. Radiological changes are non‐specific and can include ill‐defined nodular, ground glass, or consolidative changes due to migratory parasites or egg deposition with focal granulomatous change. Both acute and chronic schistosomiasis can be successfully treated with praziquantel; however, improvement of the chronic manifestations, including pulmonary hypertension, has been less convincing 5.

With increasing recognition of IgG4‐RD (including IgG4‐related lung disease), histopathological confirmation and clinico‐pathological correlation is vital. Given the diverse array of conditions that may potentially ‘mimic’ IgG4 RD, and the need for glucocorticoid therapy in the treatment in many confirmed cases, failure to exclude infective and malignant causes of elevated serum IgG4 levels may result in potentially serious consequences.

### Disclosure Statements

Appropriate written informed consent was obtained for the publication of this case report and accompanying images.
